# General practice management of depression among patients with coronary heart disease in Australia

**DOI:** 10.1186/s12875-022-01938-x

**Published:** 2022-12-16

**Authors:** Habiba Jahan, Carla Bernardo, David Gonzalez-Chica, Jill Benson, Nigel Stocks

**Affiliations:** 1 Discipline of General Practice, Adelaide Medical School, Faculty of Health and Medical Sciences, The University of Adelaide, 115 Grenfell Street, Level 8, Adelaide, SA 5000 Australia; 2 Adelaide Rural Clinical School, Faculty of Health and Medical Sciences, The University of Adelaide, 115 Grenfell Street, Level 8, Adelaide, SA Australia

**Keywords:** Depression, Coronary heart disease, General practice, Antidepressants, Gender differences

## Abstract

**Background:**

Incident depression is associated with coronary heart disease (CHD) and increased morbidity and mortality. Treatment of depression with antidepressants and psychotherapy can be beneficial for these patients to reduce the risk of further CHD events. Ongoing management of CHD and depression mainly occurs in the community, but little is known about the identification and care of patients with comorbid CHD and depression in general practice. This study explores the prescription of antidepressants for these patients by sociodemographic variables.

**Methods:**

This is an open cohort study with de-identified data based on electronic medical records of 880,900 regular patients aged 40 + years from a national general practice database in Australia (MedicineInsight). Data from 2011–2018 was used to classify patients as newly recorded CHD (CHD recorded in 2018 but not in previous years), previously recorded CHD (CHD recorded between 2011–2017) or no recorded history of CHD. Antidepressant prescribing in 2018 considered active ingredients and commercial brand names. The association between sociodemographic variables and antidepressant prescribing was tabulated according to the CHD status.

**Results:**

The proportion of current depression among patients with newly recorded CHD was 11.4% (95%CI 10.3–12.6), 10.5% among those with previously recorded CHD (95%CI 10.0–11.1) and 9.6% among those with no recorded history of CHD (95%CI 9.2–10.1). Antidepressant prescribing was slightly higher among those with newly recorded CHD (76.4%; 95%CI 72.1–80.6) than among those with previously recorded CHD (71.6%; 95%CI 69.9–73.2) or no history of CHD (69.5%; 95%CI 68.6–70.4). Among males with newly recorded CHD and depression, antidepressant prescribing was more frequent in major cities or inner regional areas (~ 81%) than in outer/remote Australia (66.6%; 95% CI 52.8–80.4%).

**Conclusions:**

Although antidepressant prescribing was slightly greater in those with newly recorded CHD compared to those with depression alone, its clinical significance is uncertain. Much larger differences in prescribing were seen by geographic location and could be addressed by innovations in clinical practice.

**Supplementary Information:**

The online version contains supplementary material available at 10.1186/s12875-022-01938-x.

## Introduction

Cardiovascular diseases, including coronary heart disease (CHD) and other heart and blood vessel disorders, remain the leading cause of death worldwide [1]. CHD alone represented 11% of all deaths and 42% of cardiovascular deaths among Australians in 2018 [2]. It is estimated that about 160 CHD events occur daily, with annual costs exceeding $2 billion in Australia. Biological and psychological factors, such as depression, play a role in the etiology, reoccurrence and morbidity associated with CHD [3–5]. Meta-analyses [6, 7] and observational studies [5] have found that depression can have deleterious effects on mortality among patients with CHD, even after controlling for potential confounders [5, 6].

Depression is more common in patients with CHD than in the general population, but estimates vary depending on the instrument used or threshold criteria for diagnosis [3, 8]. A systematic review found that among patients hospitalised for acute myocardial infarction, the prevalence of depression ranged from 10 to 46% using different assessment methods [3].

Management of depression with counselling and/or antidepressants also appears to reduce the effect of depression on mortality among patients with pre-existent CHD [9]. Patients with untreated depression have a 70–90% higher risk of dying one year after their first CHD event compared to patients either without depression or with treated depression [5, 10, 11].

In Australia, most patients with symptoms of depression will present to their general practitioner (GP) [12], who diagnoses and manages depression along with other comorbidities [13]. Whilst much has been published about GP management of depression in the community [14–16], only a few studies have addressed its relationship with comorbid CHD [17, 18]. Poor access to healthcare services, including GP and psychology services, is a major health issue in rural and regional Australia [16, 19, 20]. Still, little is known about how this affects the management of patients with comorbid depression and CHD. Furthermore, there are known associations between depression and age, sex [21] and other sociodemographic factors [22], especially in older persons [23]. These factors also can affect CHD [18, 24]. Reducing the burden of depression through early screening, as well as providing psychological and pharmacological intervention in high-risk populations can improve the overall health status of people with CHD in Australia. Indeed, GPs are ideally placed to undertake early assessment and prescribe pharmacotherapy where needed. GPs are more frequent prescribers of antidepressants than other health professionals, including psychiatrists in Australia (86% vs 10%) [13], which highlights the importance of examining their antidepressant prescribing.

Lastly, most studies investigating the management of depression among patients with CHD were undertaken in hospitals or specialised clinics/centres [25, 26]. The few studies exploring these outcomes in primary care settings were mainly conducted in the United States (USA) and Europe [12–15]. Furthermore, many of these were done with only a small number of patients (*n* = 1513) [27] or had shorter follow-up periods [5]. Therefore, we wanted to examine the care of patients with comorbid CHD and depression in general practice and explore the use of antidepressants by sociodemographic variables. Because electronic medical records (EMRs) are being used widely by researchers, especially to investigate complex associations between diseases managed in primary care [27–29], we decided to use MedicineInsight, a large-scale primary care database of longitudinal de-identified EMRs with over 2.8 million Australian patients. It was established by NPS MedicineWise in 2011 [30] and has been successfully used to explore chronic medical conditions and their associations with sociodemographic factors [31–34].

## Methods

### Data source and sample

The MedicineInsight database collects de-identified clinical information from participating general practices, varying in size, billing methods, and type of services, from all Australian states and territories (around 8% of all practices in the country). Routinely collected data includes sociodemographic (i.e. gender, year of birth, Indigenous status) and medical history data (i.e. diagnoses, reasons for consultation, immunisations, prescribed medications, laboratory results). To improve data quality, only data from practices established for at least two years and with no interruptions in data transfer greater than six weeks were included in this study [30]. Additionally, data was restricted to adults aged 40 + years considered ‘regular’ patients in the general practice (at least three consultations between 2017 and 2018, with at least one consultation in each of these two years).

### Data extraction and definitions

To identify the variables of interest, data from different fields of the MedicineInsight database from 2011 to 2018 were explored. The fields ‘diagnosis’, ‘reason for encounter’ and ‘reason for prescription’ were used to identify the diagnosis of ‘depression’ recorded by GPs [30]. The algorithm included the terms 'depression', 'depressed', 'depressive', 'depression/anxiety' or misspellings of these terms, which are likely to represent moderate to severe form of depression. Dysthymia, mood disorder and adjustment disorder were not included, as they may represent mild forms of depression.

Patients were classified as having: 1) ‘no depression’ if they did not have any record of depression during the whole study period (2011–2018), 2) ‘past depression’ if they had a diagnosis of depression in any year from 2011 to 2016 but did not have it recorded in 2017 or 2018, or 3) ‘current depression’ if they had a diagnosis of depression in 2017 or 2018. 

A similar strategy was used to identify patients with a recorded diagnosis of CHD or a procedure that represented a CHD event. Standard clinical terminology was used (e.g., angina OR heart attack OR coronary disease OR ischaemic cardiomyopathy OR myocardial infarction OR endarterectomy OR angioplasty), abbreviations, or misspellings of these terms. Patients were considered as having: 1) ‘no history of CHD’ if they did not have any record of CHD in the whole study period (2011–2018), 2) ‘previously recorded CHD’ if they had a record of CHD between 2011 and 2017, or 3) ‘newly recorded CHD’ when they had the first record of CHD in 2018.

Antidepressant prescribing in 2018 was extracted from the field ‘scripts’ using either active medicine ingredients or commercial brand names, and included the following groups, according to the Anatomic Therapeutic Classifications (ATC) system: 1) SSRIs, 2) selective noradrenaline reuptake inhibitors (SNRI), 3) tricyclic antidepressants (TCA), 4) monoamine oxidase inhibitors (MAOI), 5) other antidepressants.

The classification of the medications included in this study is outlined in Supplementary table 1.

### Data analysis

The proportion of antidepressant prescribing was calculated for all patients with ‘current depression’, according to their CHD status (no history of CHD, previously recorded CHD, newly recorded CHD). Logistic regression models were performed to analyse associations of antidepressant prescribing with patient and practice characteristics. Practice characteristics include rurality (classified as major cities; inner regional; outer regional/remote/very remote areas, with the last group representing rural areas) and the Index of Relative Socio-economic Advantage and Disadvantage (IRSAD) in quintiles. MedicineInsight provides data on rurality and IRSAD using the practice postcode. Rurality was defined using the Australian Statistical Geography Standard (ASGS), which considers population size and distance to main services [35]. IRSAD is a macroeconomic measure of relative advantage and disadvantage developed by the Australian Bureau of Statistics that summarises information about the social and economic conditions of households within an area (i.e. income, education, employment, occupation and housing characteristics) and is based on residential postcodes. A higher IRSAD score indicates a person resides in a more advantaged area (e.g. more families with high income, people in highly skilled occupations, and few families with low incomes or in unskilled occupations) [36]. Patient variables included age (40–49, 50–59, 60–69, ≥ 70 years), gender (male, female), and IRSAD quintiles. Associations of antidepressants with patient variables were adjusted for age, gender, IRSAD quintile and practice variables, while associations with practice characteristics were mutually adjusted. Marginal adjusted prediction of antidepressant prescribing in each category of the exposure variables was estimated and presented with their respective 95% confidence intervals (CI). This study also investigated whether age, gender, rurality or IRSAD affected the association between antidepressant prescribing. Therefore, multiplicative terms between those variables were included in the regression models, and when the heterogeneity of the effects was verified (*p*-value for interaction < 0.05), results were stratified and presented graphically with their 95% CI. All analyses were performed in the statistical software Stata 16.0 (StataCorp, Texas, USA), and the models considered the clustering of patients within the practice.

The independent MedicineInsight Data Governance Committee approved the study (protocol 2019–029), and the Human Research Ethics Committee of the University of Adelaide exempted it from a full review as it uses only existing and non-identifiable data.

## Results

**Fig. 1:**
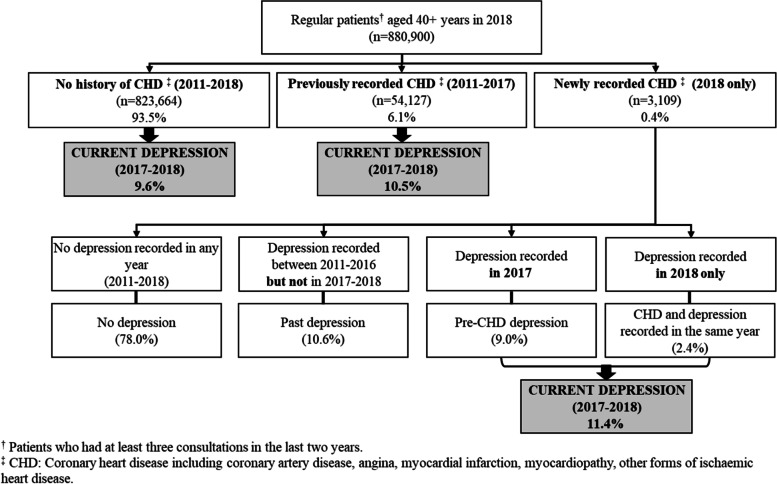
Patients’ distribution and prevalence of depression among those with no history of CHD, previously recorded CHD or newly recorded CHD in 2018

Among 1,413,971 regular patients in the database, 880,900 were 40 years or older and were included in the sample. Of these, 6.1% (95% CI 5.9–6.4) had a previously recorded CHD diagnosis, while 0.4% (95% CI 0.3–0.4) were newly recorded CHD cases. The prevalence of current depression (i.e. recorded diagnosis of depression in 2017 and/or 2018) was similar among those with no recorded history of CHD (9.6%; 95% CI 9.2–10.1), previous CHD (10.5%; 95% CI 10.0–11.1) or newly recorded CHD (11.4%; 95% CI 10.3–12.6) (Fig. 1).

The sociodemographic characteristics of those with current depression according to their CHD status (no history of CHD, previously recorded CHD or newly recorded CHD) is detailed in Table 1. Almost 60% of patients with current depression attended practices in major cities, and less than 20% in outer regional/remote areas, regardless of their CHD status. The age distribution of patients with current depression with no history of CHD varied from those with previously recorded or newly recorded CHD. In the former, 60% of patients with depression were aged < 60 years, while in the last two groups, more than 80% of those with depression were aged 60 + years.

**Table 1:** Sociodemographic distribution of regular patients with current depression by CHD status. Australia, 2011–2018

	No history of CHD			Previously recorded CHD			Newly recorded CHD			*p*-value*
	%	95%CI		%	95%CI		%	95%CI		
**Practice characteristics**										
**Rurality**										0.161
Major cities	58.3	52.2;	64.3	57.6	50.9;	64.0	56.8	48.0;	65.2	
Inner regional	28.2	22.9;	34.0	28.4	22.8;	34.7	24.7	18.6;	32.0	
Outer regional/Remote	13.5	9.9;	18.1	14.0	10.2;	19.0	18.5	11.5;	28.4	
**IRSAD Quintile**										0.912
Very high	22.3	17.7;	27.8	18.5	14.2;	23.8	20.0	14.4;	27.2	
High	17.8	13.5;	23.0	17.2	12.7;	22.8	16.2	10.2;	24.7	
Middle	23.9	19.0;	29.7	23.9	18.6;	30.1	24.0	17.5;	31.8	
Low	16.9	13.0;	21.7	18.5	14.2;	23.8	19.6	14.1;	26.7	
Very Low	19.1	14.3;	25.0	21.9	16.2;	29.0	20.2	13.8;	28.6	
**Patient characteristics**										
**Age**										0.024
40–49	32.0	31.1;	32.9	4.3	3.8;	4.9	2.1	1.2;	3.9	
50–59	28.6	28.1;	29.1	13.7	12.6;	14.9	17.2	14.1;	20.9	
60–69	20.2	19.7;	20.7	24.3	23.2;	25.5	25.0	21.3;	29.0	
> = 70	19.2	18.3;	20.0	57.7	55.8;	59.5	55.7	51.0;	60.2	
**Gender**										0.015
Male	35.9	35.0;	36.8	55.6	54.2;	57.0	49.9	45.4;	54.5	
Female	64.1	63.2;	65.0	44.4	43.0;	45.8	50.1	45.5;	54.6	
**IRSAD Quintile**										0.965
Very high	20.9	17.4;	24.8	17.4	14.0;	21.5	18.3	13.8;	24.0	
High	17.0	14.5;	19.7	16.7	13.8;	20.0	16.2	10.9;	23.3	
Middle	24.2	20.7;	28.1	23.8	19.7;	28.4	23.5	18.4;	29.5	
Low	18.5	15.5;	21.9	19.8	16.4;	23.8	20.5	15.8;	26.2	
Very Low	19.4	16.0;	23.4	22.3	18.2;	27.1	21.5	16.7;	27.3	

*CHD* Coronary heart disease, including coronary artery disease, angina, myocardial infarction, myocardiopathy, other forms of ischaemic heart disease, *IRSAD* Index of Relative Socioeconomic Advantage and Disadvantage

^*^Pearson’s chi-square test between previously recorded CHD and newly recorded CHD

Among patients with depression and a newly recorded CHD event, there were equal proportions of men and women. This contrasts with figures for previously recorded CHD (56% were men) and no history of CHD (64% were women). Considering practice and patient socioeconomic levels (IRSAD quintiles), similar patterns were observed. Among those with no history of CHD, more than 60% were from middle to very high IRSAD quintile, whilst in those with previous or newly recorded CHD, 60% were from middle to very low IRSAD.

**Fig. 2:**
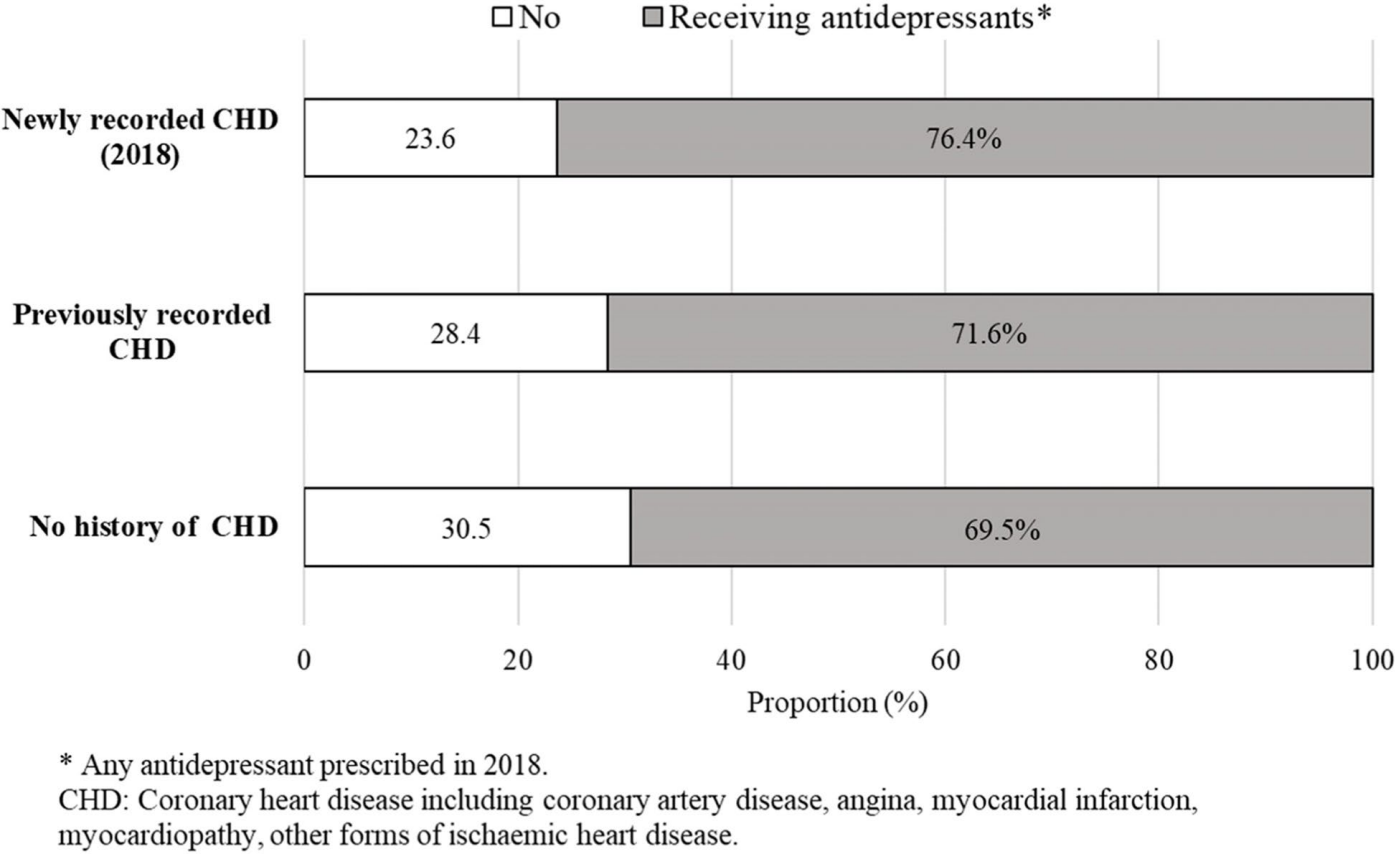
Recorded antidepressant prescribing among regular patients with current depression and no history of CHD, previously recorded CHD or newly recorded CHD in Australian general practices. MedicineInsight, 2018

Figure 2 shows that the proportion of patients with current depression recorded as being managed with antidepressants was slightly higher among those with newly recorded CHD (76.4%; 95% CI 72.1–80.6) than among those with past CHD (71.6%; 95% CI 69.9–73.2) or no history of CHD (69.5%; 95% CI 68.6–70.4).

Antidepressant prescribing recorded for patients with current depression varied according to sociodemographic characteristics and CHD status (Table 2). Patients with no history of CHD and those with previously recorded CHD attending practices in inner or outer regional/remote areas were more likely to be prescribed antidepressants than those attending practices in major cities. On the other hand, among those with newly recorded CHD, antidepressant prescribing was slightly higher in major cities, although the confidence intervals overlapped. There was no difference in prescribing for men and women with newly recorded CHD. However, antidepressants were more often prescribed to women than men if the patient had previously recorded CHD or had no history of CHD. In terms of age groups, older people were more likely to be treated with antidepressants if they had CHD in the past or no history of CHD, whilst among those with newly recorded CHD the proportion of younger and older patients receiving antidepressants was similar. Finally, patients with depression and newly recorded CHD living in the most disadvantaged areas were 25% more likely to be treated with antidepressants than those from the most advantaged areas.

**Table 2:** Adjusted proportion^a^ of antidepressant prescribing in 2018 among regular patients with current depression by CHD status and sociodemographic characteristics

	No history of CHD		Previously recorded CHD		Newly recorded CHD	
	% on antidepressants	95%CI	% on antidepressants	95%CI	% on antidepressants	95%CI
**Practice characteristics**						
**Rurality**						
Major cities	67.1	65.8;68.5	70.3	67.6;73.0	79.2	74.2;84.3
Inner regional	72.0	70.0;74.0	75.6	72.5;78.8	74.8	65.2;84.4
Outer regional/Remote	73.8	72.0;75.7	74.5	70.5;78.4	74.4	64.3;84.4
**IRSAD Quintile**						
Very high	69.1	67.0;71.2	73.5	70.0;76.9	73.4	62.9;83.9
High	69.5	67.1;71.9	72.8	69.0;76.6	69.7	60.0;79.4
Middle	71.0	69.2;72.8	71.1	67.2;75.1	80.5	72.4;88.6
Low	68.9	66.7;71.1	74.0	70.7;77.3	80.6	71.8;89.3
Very Low	68.1	65.6;70.5	71.1	66.6;75.6	79.9	70.9;88.9
**Patient characteristics**						
**Gender**						
Male	67.4	66.2;68.5	70.7	68.6;72.8	78.3	72.8;83.9
Female	70.6	69.6;71.5	74.5	72.5;76.5	76.2	70.5;82.0
**Age**						
40–49	67.4	66.3;68.5	68.3	61.5;75.1	79.5	55.3;100.0
50–59	69.1	68.1;70.2	68.7	65.0;72.4	75.2	65.5;84.9
60–69	70.8	69.7;72.0	73.8	71.1;76.4	74.8	66.9;82.6
> = 70	71.6	70.3;73.0	73.0	71.1′74.9	78.9	73.8;84.0
**IRSAD Quintile**						
Very high	68.0	66.1;69.9	70.6	65.9;75.4	66.5	51.3;81.8
High	69.1	67.5;70.7	69.8	65.9;73.6	77.4	67.1;87.7
Middle	69.5	67.9;71.0	73.2	69.8;76.6	80.9	71.1;90.6
Low	69.6	67.8;71.4	75.2	71.2;79.2	74.7	64.0;85.5
Very Low	71.1	69.4;72.8	72.4	68.6;76.1	83.1	74.5;91.7
**Total**	**69.5**	**68.6,70.4**	**71.6**	**69.9,73.2**	**76.4**	**72.1,80.6**

*CHD* Coronary heart disease, including coronary artery disease, angina, myocardial infarction, myocardiopathy, other forms of ischaemic heart disease, *IRSAD* Index of Relative Socioeconomic Advantage and Disadvantage

^a^Marginal adjusted predicted probabilities of recorded antidepressant prescribing in each category of the exposure variables. Analyses between antidepressant prescribing and patient’s characteristics were adjusted for age, gender and practice variables, while associations with practice characteristics were mutually adjusted

*P*-values were based on adjusted Wald test

**Fig. 3:**
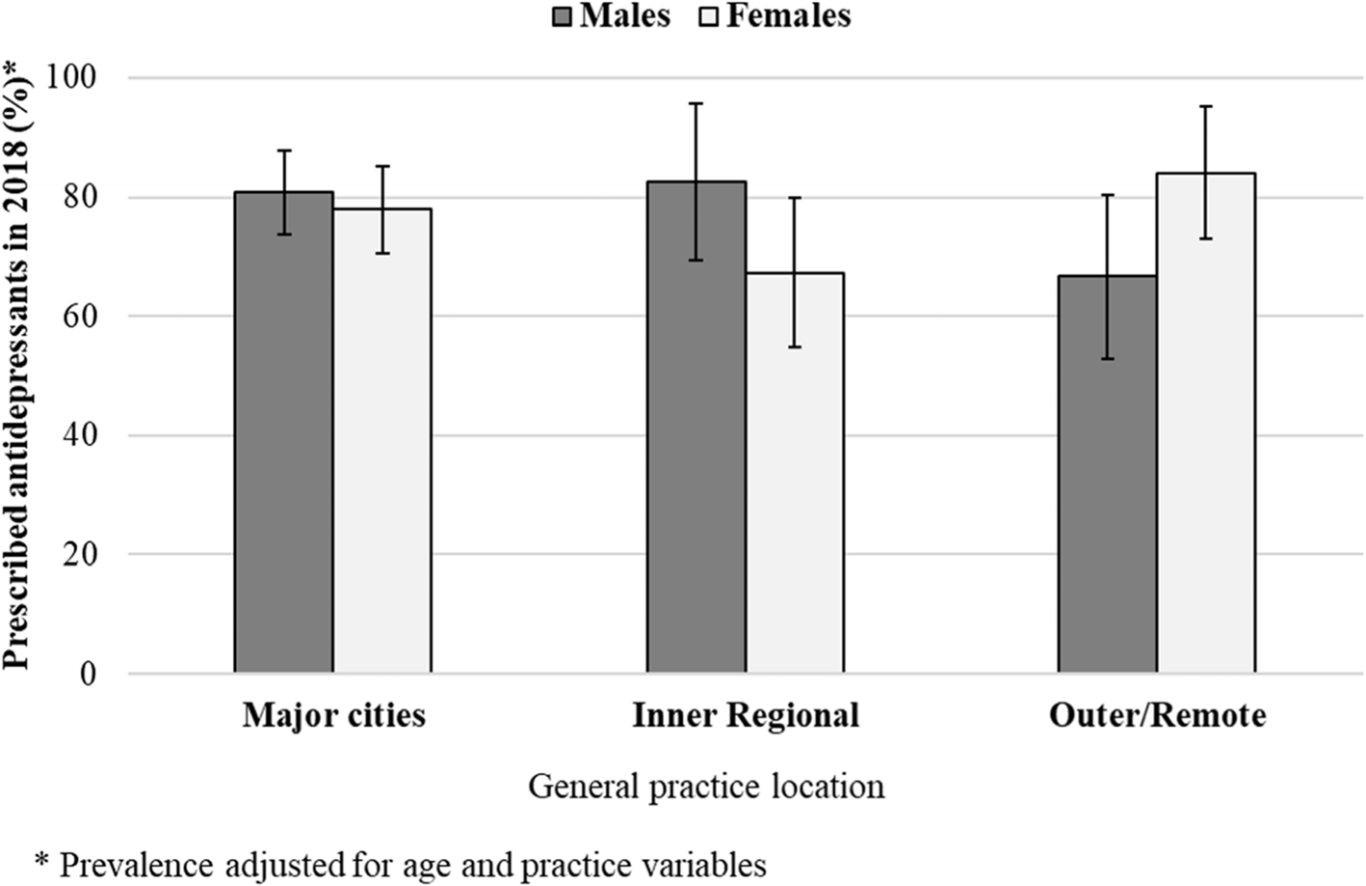
Prevalence of recorded antidepressant prescribing in 2018 among regular patients with current depression and newly recorded CHD

Additional analyses were performed to evaluate the effect of gender on the association between antidepressant prescribing and age, rurality or IRSAD. Only gender and rurality of the practice showed heterogeneity of effects. Figure 3 shows the proportion of men and women with depression and newly recorded CHD receiving antidepressants, according to rurality of the practice they visited. Men visiting practices located in outer regional/remote areas were less likely to be prescribed antidepressants than those visiting practices in major cities or inner regional areas.

## Discussion

This study has three main findings. First, the prevalence of recorded depression was not appreciably different among patients with no recorded history of CHD, previously recorded CHD or newly recorded CHD. Second, recorded antidepressant prescribing was slightly higher for patients with newly recorded CHD and co-existent (current) depression than those with past CHD. Third, patients with newly recorded CHD and current depression were more likely to be older, with similar prevalence among males and females. However, males living in outer regional/remote areas and females in inner regional areas were less likely to receive antidepressants than those living in major cities.

The prevalence of current depression among patients with newly recorded CHD was found to be 11.4%. International studies have reported that up to two-thirds of patients with acute myocardial infarction develop mild forms of depression, while major depression can be found in 15% of patients with CVD [9, 26, 37]. Although the prevalence of depression among patients with no CHD, previously recorded CHD or newly recorded CHD was slightly different, the absolute difference was small in clinical or public health terms. In comparison, world mental health surveys that were done across 17 countries where heart disease was ascertained by self-report and mental disorders were assessed with diagnostic interviews, found that the prevalence of major depression in patients with heart disease was 9.2% in the USA, 18.6% in Ukraine, 4.2% in New Zealand, 10.3% in South Africa and 4.6% in Japan [37]. Higher estimates were found in studies done in hospital settings where patients were followed up after having a CHD event with a depression screening tool. The prevalence of major depression was 18% during hospital discharge, which increased up to 37% after 3 months among survivors of acute myocardial infarction in the USA [38]. The different estimates probably reflect that most prevalence studies identified depression in hospital settings [3, 9, 39], which is not the same as clinical pathways in primary care. Methodological differences, demographic variations, comorbidities, tools used for identifying depression, length of follow-up, and access to hospital care may also account for different estimates across studies [6, 26, 39–43].

Many studies have reported that depression is associated with poor cardiac outcomes [5, 6, 26, 39, 42, 44]. Guidelines from the Royal Australian and New Zealand College of Psychiatrists recommended a combination of psychotherapy and pharmacotherapy for the treatment of moderate to severe depression [45]. There has been a gradual increase in the use of antidepressants in Australia [46], the United Kingdom [47], the USA [48] and New Zealand [49]. Given this evidence, one might expect that GPs would be more likely to prescribe antidepressants to patients who had CHD in the past or had survived incident CHD. The results of this study suggest that there were only small differences in the rate of antidepressant prescribing among patients with depression, with or without a history of CHD. This result might be explained, in part, by either inadequate recognition of depression. For instance, fatigue or insomnia can coexist with depression and CHD, or a patient may have mild depression that may not warrant antidepressants, or potential concerns regarding the safety of antidepressants by GPs or patients [50]. However, more recent studies have proven the safety of cardioprotective antidepressants (SSRI, SNRI) [43, 51]. Lastly, patients presenting to GPs months after incident CHD might have recovered from reactive depression without intervention.

Examination of the sociodemographic characteristics of patients with current depression and newly recorded CHD found that 55.7% were aged > 70 years, with similar proportions of males and females. These findings are contrary to the MINDSMAPS meta-analysis, which showed that women tend to have a higher incidence of depression than men according to both diagnostic interview and questionnaire studies post-myocardial infarction, which is the major component of CHD [52]. On the other hand, this discrepancy might indicate that our analysis describes a more accurate pattern in Australia, which has been previously described in a South Australian study (*n* = 1563) performed in rural settings, where no gender differences were found on measures of psychological distress, anxiety or depression [53].

An additional analysis was run in our study to look at the association between rurality and the prescription of antidepressants. Men visiting practices located in outer regional/remote areas were less likely to be prescribed antidepressants than those visiting practices in major cities or inner regional areas. This might indicate poor access / uptake of healthcare services by men in rural areas where there is a general shortage of healthcare professionals, including GPs, psychologists, and allied health practitioners [54]. In inner regional Australia, fewer women than men received antidepressants for depression post-CHD, possibly due to lower incomes, fewer employment opportunities and lower educational attainment [12, 31], making them more vulnerable than men to the effect of depression and comorbid CHD. Given the size of these prescribing differences and potential adverse clinical outcomes in those not being treated, it will be important to institute policy and clinical pathway changes to improve antidepressant prescribing rates in these populations. Brief motivational care intervention was found to reduce mental health severity and substance use disorder in rural Australia than usual care [55]. Telehealth services have been more widely utilised since the Covid-19 pandemic to address different health issues, including mental health illness [56], and they can be an effective way to deliver routine follow-up in patients after a CHD for screening as well as psychoeducation.

### Strengths and limitations

A strength of this study is the large number of adult patients contributing data from practices from all Australian states and territories. The study presents real-world data of the Australian population attending general practice seeking care for the conditions of interest. Also, the terms “depression”, “depressed” and “depression/anxiety” were used for the definition of depression, but not “adjustment disorder/mood disorder/dysthymia”, which makes the result more likely to identify moderate or severe depression. However, it is difficult to determine the severity of depression using EMR since progress notes and depression assessment tool scores are not extracted.

As a limitation, the diagnosis of depression was based on what the GP had recorded in specified fields of the EMR. However, the data extraction did not include the progress notes, which often contain additional clarifying information and depression assessment tools. Moreover, angina was one of the investigated CHD conditions. However, the diagnosis of angina can be recorded in the clinical history without any confirmatory evidence of CHD. Nonetheless, MedicineInsight data extraction algorithms for conditions such as depression, anxiety and diabetes have shown specificity, positive predictive values, and negative predictive values equal to or higher than 0.9 compared to the original medical records at the practice (i.e. including progress notes) [29]. In addition, TCA, SSRI, and atypical antidepressants were included in the analysis, but these can also be prescribed for indications other than depression, although these numbers would be expected to be small. This study only captures prescriptions that were written by the GP rather than prescriptions that were dispensed or taken. Antidepressants’ dose augmentation, switchover or tapering were also not explored, which could be a topic for future studies. Although prescriptions written in hospitals or by psychiatrists were not explored, the actual number would be expected to be relatively small given the difficulty in accessing public or private psychiatric services in Australia, and the limited long-term community prescribing done by hospital doctors. Finally, MedicineInsight does not link patients across different practices, potentially duplicating patient information when they attend multiple sites. However, the estimate is that only 4% of patients visit more than one practice.

## Conclusion

Although the identification of depression among patients with CHD in Australian general practice is consistent with international studies, more could be done to ensure a consistent approach to depression diagnosis. In Australia, there are funded avenues (e.g. Chronic disease management plans) [57] to undertake screening for depression in patients with chronic disease. Further research may identify mechanisms to incorporate them into routine practice. Disparity in access to health care in rural and remote Australia is well recognised, but addressing the identified pharmacological treatment gaps by sex and area of residence will be more challenging. The success of telehealth in Australia during the Covid-19 pandemic maybe one pathway to readdress these health inequalities.

## Supplementary Information

**Additional file 1: Supplementary Table 1.** Antidepressants included in the study.

## Data Availability

The data used in this study was obtained from NPS MedicineWise MedicineInsight under license for the current study, so it is not publicly available. However, other researchers may be able to access the data if approval is granted by the MedicineInsight Data Governance Committee. Data access enquiries can be directed to medicineinsight@nps.org.au.

## Abbreviations

ABS: Australian Bureau of Statistics
ATC: Anatomic Therapeutic Classifications
CHD: Coronary Heart Disease
CI: Confidence Interval
EMR: Electronic medical records
GP: General Practitioners
IRSAD: Index of Relative Socioeconomic Advantage and Disadvantage
MAOI: Monoamine oxidase inhibitors
NPS: National Prescribing Service
SNRI: Selective noradrenaline reuptake inhibitors
SSRI: Selective serotonin reuptake inhibitors
TCA: Tricyclic antidepressants
USA: United States of America
