# Supplementary material for: General practice management of depression among patients with coronary heart disease in Australia
Source: BMC Prim Care. 2022 Dec 16;23:329. doi: 10.1186/s12875-022-01938-x (PMC9755773; doi:10.1186/s12875-022-01938-x)
Supplement: Supplementary file 1 — Additional file 1: Supplementary Table 1. Antidepressants included in the study. [file 12875_2022_1938_MOESM1_ESM.docx]

| Supplementary table 1. Antidepressants included in the study. | | | | |
| --- | --- | --- | --- | --- |
| **SSRI** | **SNRI** | **TCA** | **MAOI** | **OTHER** |
| Citalopram | Desvenlafaxine | Amitriptyline | Phenylzine | Agomelatine |
| Escitalopram | Duloxetine | Clomipramine | Moclobemide | Bupropion |
| Fluoxetine | Venlafaxine | Dothiepin |  | Mianserin |
| Fluvoxamine |  | Doxepin |  | Mirtazepine |
| Paroxetine |  | Imipramine |  | Reboxetine |
| Sertraline |  | Nortriptyline |  | Tranylcypromine |
|  |  | Trimipramine |  | Vortioxetine |
